# Is There a Downside of Job Accommodations? An Employee Perspective on Individual Change Processes

**DOI:** 10.3389/fpsyg.2017.01536

**Published:** 2017-09-20

**Authors:** Julia M. Kensbock, Stephan A. Boehm, Kirill Bourovoi

**Affiliations:** ^1^Department of Economics and Business Administration, University of Duisburg-Essen Essen, Germany; ^2^Center for Disability and Integration, University of St. Gallen St. Gallen, Switzerland

**Keywords:** aging workforce, job accommodation, workplace accommodation, disability, impairment, interpersonal conflicts, organizational change

## Abstract

By modifying the work environments, work routines, and work tasks of employees with health restrictions, organizations can effectively help them continue to perform their jobs successfully. As such, job accommodations are an effective tool to secure the continued employment of aging workers who develop disabilities across their life span. However, while accommodations tackle health-related performance problems, they might create new challenges on the part of the affected employee. Building on the organizational change and accommodations literatures, we propose a theoretical framework of negative experiences during accommodation processes and apply it to qualitative data from group interviews with 73 manufacturing workers at a German industrial company who were part of the company's job accommodation program. Although problems associated with health-related impairments were mostly solved by accommodation, affected employees with disabilities reported about interpersonal problems and conflicts similar to those that typically occur during organizational change. Lack of social support as well as poor communication and information were raised as criticisms. Furthermore, our findings indicate that discrimination, bullying, and maltreatment appear to be common during accommodation processes. To make accommodation processes more successful, we derive recommendations from the organizational change literature and apply it to the accommodation context. We also emphasize unique characteristics of the accommodation setting and translate these into practical implications.

## Introduction

Organizations are confronted with a growing number of persons with physical impairments and disabilities, and many of these health impairments are due to individual aging processes (WHO, 2011; Boehm and Dwertmann, 2015). A primary trigger of this development is a societal phenomenon often referred to as *demographic change* (Dychtwald et al., 2004; Kulik et al., 2014): Due to the combined effects of low birthrates and increased longevity, the average ages of entire nations as well as their workforces are rising. To maintain the long-term employability of individuals in organizations is a primary corporate challenge of our time (Bal et al., 2013; Zacher and Yang, 2016).

In addressing this challenge, the present study raises the question: How can workplaces be accommodated to enable employees with disabilities to work in ways that both add value for a firm and are satisfying for an affected individual? Job accommodations encompass “modifications in the job, work environment, work process, or conditions of work that reduce physical and social barriers” (Colella and Bruyère, 2011, p. 478). Thus, accommodations establish new working conditions and equal opportunities for a wide range of individuals with health restrictions. In this study, we apply a broad definition of disability as being an “umbrella term for impairments, activity limitations and participation restrictions, referring to the negative aspects of the interaction between an individual (with a health condition) and that individual's contextual factors (environmental and personal factors)” (WHO, 2011, p. 4). Although disabilities can affect individuals of all age groups, there is a high correlation of age and disability (Colella and Bruyère, 2011; WHO, 2011). That is, older workers tend to experience more significant health limitations, putting their further employment at risk. As such, our research question entails important implications for organizations dealing with the challenge of an aging workforce in general and the increasing prevalence of disabilities in particular.

In answering the above-noted research question, the present study makes three important contributions to the literature. As a first contribution, we seek to shed light on the experiences of accommodation recipients during the actual accommodation process and thus expand the focus from an *accommodation requester's view* to a *recipient's view*. To date, accommodation research has been approached mainly from the view of the employing organization or from the perspective of colleagues working with a person with a disability. These streams of research have provided important insights into the preconditions that increase the likelihood of an accommodation being granted (Florey and Harrison, 2000) and the requirements under which colleagues tend to perceive accommodations to be justified (Colella, 2001). However, there has been surprisingly little research from the perspective of the primary actors in the accommodation process, i.e., employees with disabilities themselves (Balser and Harris, 2008). As one important exception, Baldridge and colleagues (Baldridge and Veiga, 2001, 2006; Baldridge and Swift, 2013, 2015) as well as Davison et al. (2009) considered the perspective of employees with disabilities and systematically examined their tendency to *request* accommodations. Specifically, these studies focused on identifying factors that prevent employees with disabilities from requesting future job accommodations. While these studies constitute important steps toward understanding the psychological processes associated with *accommodation requests*, scholars have largely neglected the question of what employees actually experience *after* the accommodation has been requested and granted, and the present study aims at closing that gap.

As a second contribution, the present study seeks to *provide insights regarding the challenges associated with job accommodations*, with the goal to understand critical success factors and to derive recommendations about how to improve such processes within organizations. Consequently, the present study focuses on examining *negative* experiences among accommodation recipients in a systematic, differentiated manner. Notably, the bulk of prior research has primarily focused on the *benefits* of job accommodations for employees and organizations. That is, many studies assume that after the granting of an accommodation, the situation improves for the affected employees, since their health issues are potentially solved and their further employment is secured (Schartz et al., 2006; Colella and Bruyère, 2011). We agree that accommodations are an indispensable opportunity to ensure the employability of older people developing disabilities throughout their working lives and to increase the productivity of employees and organizations (Solovieva et al., 2011; Solovieva and Walls, 2013). At the same time, we argue that once granted, accommodations also lead to novel and unexpected challenges for employees, especially new interpersonal problems and team conflicts.

From a theoretical point of view, we use two major streams of research that support our focus on negative experiences during job accommodations. First, we build upon the large stream of *job accommodation, aging, and disability research*. Here, prior studies show that when thinking about *requesting* job accommodations, employees with disabilities fear serious psychological and social consequences that prevent them from asking for an accommodation (Baldridge and Veiga, 2001, 2006; Baldridge and Swift, 2013, 2015). Moreover, negative past experiences in requesting accommodations affect the likelihood of individuals requesting future accommodations (Davison et al., 2009). These findings prompted Colella and Bruyère (2011) to ask whether “these concerns on the part of people with disabilities [are] justified?” (p. 479), which echoes the need to apply a more fine-grained perspective on the negative experiences of employees during accommodation processes. In addition, studies in the domain of aging clearly show that older employees are frequently confronted with negative attitudes and discriminatory behavior against them, stemming from negative stereotypes on the part of coworkers and supervisors (North and Fiske, 2012; Bayl-Smith and Griffin, 2014; MacDonald and Levy, 2016). Given that most of these stereotypes refer to older workers being less productive and adaptive (e.g., Abrams et al., 2016), it seems likely that employees with all kinds of health restrictions are confronted with similarly negative experiences at work.

Second, the *literature on organizational change* has traditionally focused on employees' negative experiences during change processes (e.g., Coch and French, 1948; Paterson and Cary, 2002; Kiefer, 2005). As a central assumption, in the present study, we suggest that accommodation processes can be understood as individual-level change processes. That way, we posit that many of the employees' experiences during accommodation processes can be compared to experiences during organizational change processes, allowing for a transfer and combination of extant research findings. Overall, shedding light on the specific negative experiences and reactions of accommodation recipients enables an enhanced understanding of critical success factors for job accommodation processes. These insights should be helpful in preventing detrimental consequences for the affected individual and, finally, the employing organization.

As a third and final contribution, the cultural and organizational context of the present study allows us to advance existing job accommodation literature by *complementing the US-based studies* that to date have characterized research on workplace accommodations. While it is specified in the Americans with Disabilities Act (ADA) that accommodation requests are typically raised by employees themselves, accommodation processes might run differently in other countries. In Germany, where this study was conducted, every employee with an official disability status has a legal right to receive workplace accommodations (§ 81 SGB IX^1^)—however, it is not officially defined *who* initiates the workplace accommodation (e.g., the employee, the employer, or a third party such as the work council). In the context of this study, accommodation requests are “imposed from the outside,” i.e., by supervisors. Specifically, supervisors initiate job accommodations in response to the fact that employees are unable to meet performance requirements owing to his or her disability. In our view, this institutional difference might impact findings on accommodations' effectiveness, since employees might perceive externally imposed accommodations more negatively.

Taken together, this study seeks to: (1) direct attention to the accommodation recipients' perceptions *during* and *after* the actual accommodation; (2) shed light on the downsides of job accommodations in order to derive success factors helping organizations to effectively implement their accommodation processes; and (3) internationalize the accommodation literature by investigating processes in a non-US setting that are not requested by employees. To obtain such comprehensive insights into accommodation recipients' experiences, we apply a qualitative approach. Using the method of template analysis (King, 1998), we develop a coding framework based on theoretical insights from both the organizational change and accommodation literatures and apply it to interview data from accommodation recipients of a large German industrial company.

## Theoretical framework

### Accommodations as individual-level change processes

The notion of change, which is an integral part of the accommodation concept, can take the form of a “change in duties, a change in a valuable commodity, a change in the physical conditions of work, a change in the tools of work, a change in resources available to coworkers, or even a change in location” (Colella, 2001, p. 101). Although we propose that organizational change processes and job accommodation processes share important similarities, findings from the organizational change literature cannot be entirely transferred to the accommodation context. An important difference concerns the level of analysis. In contrast to organizational-level change processes, job accommodation processes primarily occur at the individual level. That is, although colleagues working directly with an accommodation recipient are often also affected by a change, job accommodation processes primarily affect single employees and their immediate work environments. By contrast, organizational-level change processes typically affect many employees simultaneously. Furthermore, the reason for an accommodation is an individual-level problem, that is, an employee's health restriction rather than an organizational-level issue or a management decision. Despite these different reference points, we posit that organizational change processes (as *collective change processes*) and accommodation processes share three important features that allow us to understand accommodations as *individual-level change processes*.

First, both organizational change processes and accommodation processes are characterized by an *intentional goal to approach a challenge/an existing problem and to achieve an improved future state* (Beckhard and Harris, 1987). Organizational change can be directed at various corporate challenges (e.g., business acquisitions, process improvements, technology changes; Smith, 2002). Similarly, job accommodation processes derive from a situation that is in some way problematic, i.e., an employee's health impairments interfere with the performance expectations of his or her job. Accommodation processes thus aim to create a work environment in which employees can perform key functions of their jobs and can receive the same benefits of employment as others (Vernon-Oehmke, 1994).

Second, from the accommodation recipient's perspective, both processes share the *novelty* associated with the changed workplace situation—a feature individuals often perceive as threatening and harmful (Rafferty and Griffin, 2006). In both processes, employees are required to adapt to a new working environment, including potentially changed behaviors, duties, locations, or colleagues (Holt et al., 2007; Oreg et al., 2013). Likewise, job accommodations often require employees to adapt to new working situations, which also involve novel tasks or skills.

Third, both process types have a strong *affective significance* for individuals. In line with affective events theory (Weiss and Cropanzano, 1996), work-related processes have the potential to elicit intensive affective reactions in employees. Change processes are also interpreted as work events that provoke various affective reactions including stress, anxiety, or resistance, which—in turn—influence work attitudes such as job satisfaction or turnover intention (e.g., Ashford, 1988; Kiefer, 2005). Similarly, for accommodation recipients, the situation of being impaired in their jobs and being dependent on their employer's help can certainly be a profound landmark in their work lives, especially for individuals who acquired their disability during their employment. Therefore, it can be assumed that the perception of the accommodation process might also exert a critical influence on affected employees' wellbeing. Owing to these commonalities, we propose that accommodation experiences should be perceived and analyzed similarly to organizational change experiences and that they might elicit affective reactions comparable to typical reactions to change.

### Stakeholders in the accommodation process

Job accommodations are inherently social processes that influence and are influenced by other actors in the social environment surrounding an accommodation recipient (Gates et al., 1998; Gates, 2000). Thus, we suppose that many conflicts and problems experienced during the accommodation phase will be interpersonal. Besides the accommodation recipients themselves, several parties are involved in a typical accommodation process.

To begin with, the affected employees' *coworkers* may be directly affected by the change (García et al., 2005). Such interpersonal problems might stem from feelings of distributive injustice due to the differential treatment a single person in a group is provided when receiving an accommodation (Colella, 2001). An accommodation may be perceived as unfair by coworkers for various reasons (Paetzold et al., 2008): First, it may seem that the accommodation recipient's job becomes easier (reducing his or her inputs) while the outcome remains the same as that of others. Second, coworkers might feel that their own inputs become higher (more difficulty, inconvenience, stress) through a colleague's accommodation. Third, coworkers sometimes view accommodations as valuable and desirable outcomes given to another person but not to oneself (e.g., an ergonomic chair). Fourth, scarce resources that could also be used for other purposes might be spent for job accommodation purposes. This phenomenon is also known from the aging literature—individuals often experience a sense of “resource tension” (North and Fiske, 2013, 2016), meaning that younger versus older individuals compete for scarce resources such as job opportunities (MacDonald and Levy, 2016). The perception of unfairness by coworkers is thought to be stronger if employees work together in very interdependent ways (Colella, 2001). In such situations, an accommodation and the related job easing for one employee can cause a direct deterioration for others, since they must often take over especially difficult or exhausting elements of this employee's job. Another difficulty exists for individuals whose health impairments are not directly visible. When the reason for a job accommodation is invisible or unclear, coworkers tend to believe that the person might “fake” a health-related problem, calling into question the reason for an accommodation (Colella, 2001; Paetzold et al., 2008). An additional source of conflict between accommodation recipients and their coworkers may be the perception that a workgroup's performance is weakened by having employees with health restrictions in the team. Especially when performance is measured or even rewarded at a team level, tensions between coworkers may arise (Paetzold et al., 2008).

Besides coworkers, *supervisors* or *managers* are primary stakeholders in the accommodation process (Gates, 2000) as they are in charge of organizing the implementation of a change and are directly interacting and communicating with accommodation recipients. Supervisors might have strong concerns about employees with disabilities for several reasons. First, similar to coworkers, they might think that these employees are incapable of high performance and therefore lower the workgroup's overall performance. Indeed, prior research has shown that supervisors often perceive employees with disabilities as helpless and dependent (Baron and Neuman, 1996). Second, owing to these employees' disability, age, or health status, supervisors might feel that these employees are different from themselves (demographic dissimilarity; Turban and Jones, 1988), worsening their relationship quality with individuals with disabilities (Colella and Varma, 2001; Dwertmann and Boehm, 2016). Third, job accommodations might in many cases run counter to the business objectives that supervisors are pursuing, i.e., cost effectiveness or operating efficiency, a contradiction that might create further reservations toward employees with disabilities.

Finally, there may be other stakeholders in the accommodation process, especially in large organizations. Here, supervisors often do not organize the accommodation process on their own, but receive support from *specialized departments*, such as HR departments or company physicians (Colella and Bruyère, 2011). For them, accommodations typically generate additional work, possibly leading to negative feelings toward an employee with a disability.

### An a priori model of potential experiences during the accommodation process

**Figure 1:**
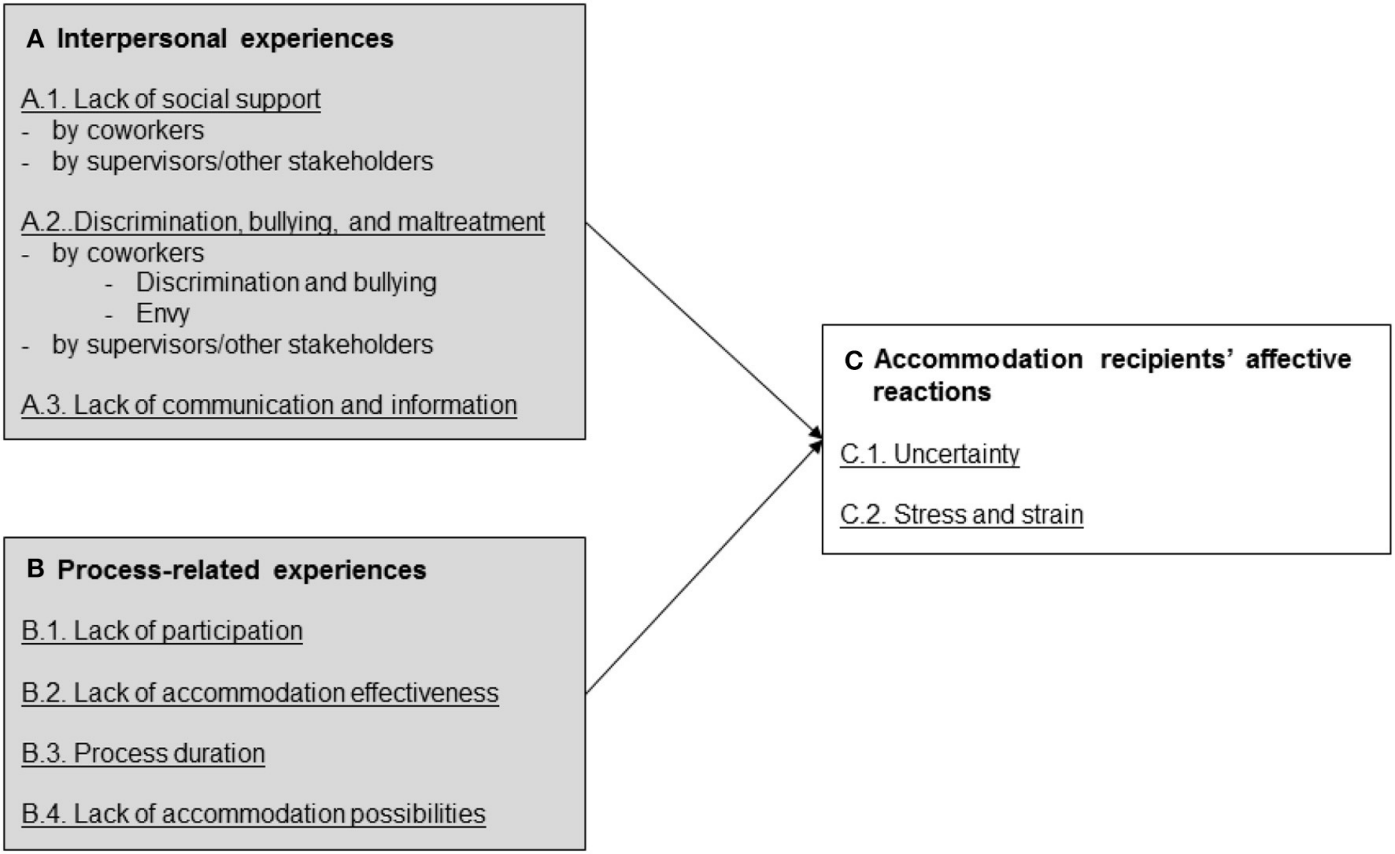
Model of negative experiences during the accommodation process.

As described above, we assume that accommodation processes share some important characteristics with general change processes, which allows us to transfer knowledge from organizational change to develop an a priori model of experiences during accommodation processes. In doing so, we build on Oreg et al. (2011) review of organizational change in which the authors introduce a comprehensive framework describing individuals' *change reactions* (affective, cognitive, and behavioral reactions to change), and also *change antecedents* triggering these reactions (e.g., characteristics of the change recipient or the internal change context). In the present study, we draw upon and extend the framework by Oreg et al. (2011) to introduce a model of experiences during the accommodation process (see Figure 1).

As outlined above, the present study primarily seeks to identify recipients' negative experiences during accommodation processes. Although organizational change processes are sometimes accompanied by positive employee reactions such as satisfaction (Jones et al., 2005) or commitment (Walker et al., 2007), that research focus is in line with the majority of studies associating change processes with various forms of psychological distress (Kiefer, 2005; Oreg et al., 2011). For the accommodation context, we propose that most of these negative experiences stem from the *absence of supportive conditions* during the process. A large proportion of past research on organizational change has identified such supportive conditions in the organizational environment that are critical for change success, including change participation (Bordia et al., 2004), communication (Lewis and Seibold, 1998), or trust in management (Morgan and Zeffane, 2003). This indicates that the absence of such supportive conditions will put change success at a risk. Similarly, from the accommodation recipient's perspective, the lack of such supportive boundary conditions will probably cause individual problems and conflicts during the accommodation phase and will likely provoke negative affective reactions. While our theoretical model focuses on negative experiences, our qualitative analysis also considers the positive perceptions of accommodation recipients in order to provide a holistic picture of accommodation experiences.

#### Negative interpersonal experiences during the accommodation process

The interpersonal experiences presented might stem from interactions with the different stakeholders described above, i.e., coworkers, supervisors, and specialized departments.

##### Lack of social support

Social support can be defined as “the availability of helping relationships and the quality of those relationships” (Leavy, 1983, p. 5). In general, social support is a powerful resource that buffers stress reactions (Viswesvaran et al., 1999) and helps an employee to manage work demands (Lysaght et al., 2012). The importance of social support has been emphasized in the organizational change literature and in studies on disability, aging, and accommodations alike. That is, during organizational change, social support shown by coworkers and supervisors/further stakeholders is a critical success factor for change initiatives (e.g., Eby et al., 2000). Likewise, for employees with disabilities, Baumgärtner et al. (2014) show that social support is positively related to job performance, especially for individuals with low self-efficacy (Baumgärtner et al., 2014). In the same manner, receiving social support from both coworkers and supervisors is crucial for the satisfaction and well-being of older employees, since it can buffer negative effects of age discrimination (MacDonald and Levy, 2016). In the accommodation context, social support can be provided by all the different stakeholders involved in the process. In workgroups, support and cooperation are important success factors for the implementation of an accommodation (Colella, 2001). Gates (2000) also suggests that supervisor support is essential for accommodation recipients and thus, if missing, a potential source of perceived problems.

##### Discrimination, bullying, and maltreatment

Employees with disabilities tend to belong to a minority in the workforce (Lengnick-Hall et al., 2008) who are confronted with comparable challenges as other marginalized groups (Ruggs et al., 2013). In particular, their disability makes them susceptible to stereotyping and stigmatization (Moore et al., 2011). This is also true for older employees in general who are confronted with negative age-related stereotypes and discrimination (e.g., North and Fiske, 2012). Given such stigmatization and the detrimental implications job accommodations might have on their colleagues, as outlined above, it seems likely that accommodation recipients are not perceived as very desirable team members (Miller and Werner, 2007). This might lead to the devaluation and exclusion of affected employees from workgroup activities (Stone and Colella, 1996). One specific phenomenon we also expect to observe in this context is that accommodation recipients might be confronted with expressions of *envy* from their coworkers. From a distributive justice perspective, Colella (2001) argues that coworkers often perceive others' accommodations as *unequal treatment* and as *unfair* because they imply more favorable working conditions.

In addition to avoidance and exclusionary behavior, we expect that coworkers' reservations against accommodation recipients also give rise to more overt hostile interpersonal behavior including open conflicts, discrimination, or bullying. Especially bullying seems likely in an accommodation situation, as members of minority groups are frequently victims of discrimination (Fine and Asch, 1988; Green et al., 2005), particularly if they have a disability that makes some accommodation necessary (Baldridge et al., 2015). Moreover, according to Salin (2003), changes to the status quo, such as workgroup composition changes but also more general organizational change processes, can serve as a trigger of bullying. Finally, increasing the number of employees with disabilities in a team also means increasing the workgroup's diversity—which, in turn, has also been shown to increase the incidence of aggressive workplace behavior owing to difficulties in communication, mutual stereotyping, and social categorization (Van Knippenberg and Schippers, 2007). Besides the discrimination and bullying conducted by coworkers, hostile behavior from supervisors is a common phenomenon known in literature (*vertical aggression*; e.g., Cortina et al., 2001). One possible explanation is that a perceived power imbalance is a prerequisite for bullying, which is especially a problem for employees belonging to minority groups (Salin, 2003). We therefore assume that accommodation recipients are likely to perceive discrimination, maltreatment, or bullying not only from coworkers but also from supervisors and other authorities in the organization.

##### Lack of communication and information

Another well-known success factor for organizational change is the communication and information about the change process on the part of the management or superiors (Lewis and Seibold, 1998; Elving, 2005). Providing employees with change-related information can help them to feel better prepared and better able to deal with a change (Bordia et al., 2004). Indeed, systematic communication has been shown to reduce uncertainty during organizational change (Schweiger and DeNisi, 1991) and to increase employees' perceived procedural justice, trust, and commitment (Gopinath and Becker, 2000).

Likewise, accommodation recipients should receive reasonable information about change processes from supervisors and other responsible stakeholders. Communication should keep a recipient updated at any point of the accommodation process and should include information about *what* the next steps are in the accommodation process, *when* these steps will be performed, *how* the accommodation will be implemented, and *who* the responsible contact person is for the process. In case this information is insufficient, this should be perceived as a considerable negative aspect of the accommodation by the affected employees.

#### Negative process-related experiences during the accommodation process

Beyond these interpersonal problems and conflicts, there are certain negative experiences inherent in the accommodation process itself. These refer to unique circumstances we expect to accompany the accommodation process in organizational practice (Solovieva and Walls, 2013).

##### Lack of participation

Participation refers to the extent of accommodation recipients' involvement during the process, especially with regard to planning and implementing the individual change (cf. Oreg et al., 2011). Having control over the process, i.e., having the opportunity to raise one's voice and being sincerely listened, should increase the perceived procedural justice of accommodation recipients (Martin et al., 2005). Moreover, participative decision-making is associated with reduced levels of physical and psychological stress (Bordia et al., 2004), and increased perception of control (Sagie and Koslowsky, 1996). In the case of workplace accommodations, seeking accommodation recipients' input during the process increased their satisfaction with the accommodation (Balser and Harris, 2008). Moreover, the feeling of actively shaping one's own career increases life satisfaction, especially of employees with disabilities (Santilli et al., 2014). As a consequence, we assume that a lack of participation during the accommodation process can result in negative experiences for affected employees.

##### Lack of accommodation effectiveness

Job accommodations aim at eliminating (or significantly reducing) difficulties associated with the health problems in everyday work. However, just like organizational change processes that are not always successful (Reichers et al., 1997), job accommodations might not reach their intended effect but might be perceived as ineffective (Solovieva and Walls, 2013). For instance, for an employee suffering from back problems who cannot carry out overhead work, an accommodated job involving bending over will not ease day-to-day work. Due to organizational constraints, however, it will sometimes be impossible to entirely solve the existing problem through job accommodations. From the perspective of an accommodation recipient, however, the perception that “accommodations aren't helping” (Solovieva and Walls, 2013, p. 203) can be expected as a central issue in the process.

##### Process duration

Another potential problem associated with organizational constraints can be the length of the process. Especially for severe health problems, providing a job accommodation can take a long period of time, for instance owing to organizational measures to be followed or financial resources that must be provided. Such delays might appear burdensome for employees and might hinder the process from being judged as successful.

##### Lack of accommodation possibilities

Employers report that a lack of accommodation possibilities is a common reason for not granting accommodations to employees (Solovieva and Walls, 2013). Indeed, most organizations are not geared to provide many workplace accommodations. Especially private enterprises operating in competitive markets often depend on their employees' flexibility in order to be able to quickly adapt to external circumstances. Adapting workplaces to individual employees' needs reduces this flexibility (e.g., job rotations). Therefore, accommodation requests may pose a challenge and considerable financial effort to such companies and in many cases, providing accommodations is no simple undertaking—presumably a severe disadvantage from an employee perspective.

#### Accommodation recipients' negative affective reactions

We suppose that the affective reactions mentioned below are consequences of the interpersonal and process-related experiences introduced above.

##### Uncertainty

Change processes are often accompanied by aversive feelings of uncertainty and anxiety (Ashford, 1988; Bordia et al., 2006). During the accommodation phase, employees often do not know how their future working situation will look like and whether an accommodation will lead to an improvement. Uncertainty can even take the form of change-related anxiety arising from an actual or perceived threat of loss (Paterson and Cary, 2002). Within accommodation processes, perceived threats of loss might include being transferred to another position, losing one's previous coworkers; salary reduction due to alterations in job design, etc. Moreover, job-related know-how and skills often cannot be transferred to a new work environment, which might result in a loss of prestige, reputation, and personal resources.

##### Stress and strain

Organizational change processes can be seen as disruptions in work life and thus stressful life events (Cartwright and Cooper, 1993). The increased stress levels during organizational change also arise from uncertainty over the future (Ashford, 1988). For the context of workplace accommodations, we propose that stress and strain will be consequences of the feeling of uncertainty on the one hand. On the other hand, interpersonal problems and conflicts during an accommodation could be direct causes of stress and strain. Especially, social stressors including discrimination, bullying, and maltreatment are likely to be associated with higher stress levels (Hansen et al., 2006).

## Method

### Organizational setting

Our study was conducted in a large manufacturing plant (15,000 employees) of a German industrial company. Throughout the study process, we took the following steps in order to ensure that all ethical research standards were fulfilled. First, the study was carried out in accordance with the Declaration of Helsinki (1964) and its later amendments. Second, our approach followed the official recommendations of the Association of German Professional Psychologists. Third, we obtained the approval of the company's work council for conducting this study and closely worked together with the data protection officer in designing this study. A strict data protection agreement was signed that closely regulated all data collection, storage, analysis, and reporting procedures. Since, in the business context, work council approval is comparable to the evaluation of a university ethical committee, further ethical approval was not required for this study in accordance with the national and institutional guidelines.

Workers in this plant were on average 40.4 years old, 89.1% were male; 13.8% of manufacturing staff had some kind of job-related health restrictions diagnosed by a (company) physician, and 4.4% had an official German disability ID documenting a disability status. The manufacturing tasks are mostly executed by teams of 8–12 employees. The team members typically rotate through the different jobs performed by the team on an hourly basis, i.e., every employee works at up to eight different workstations during one workday. Owing to this interdependent work organization, employee job flexibility is crucial for enabling job rotation.

As a result of an aging workforce and a growing number of physical or mental health problems in this organization, the company established a systematic process to deal with manufacturing employees who fail to achieve expected standard performance on the production line. The primary objective of this process is to realize an individual workplace accommodation in order to increase individual work productivity while maintaining job rotation; ideally, employees should be enabled to reach the standard performance of non-impaired workers again. Typically, the accommodation procedure is initiated by a supervisor in response to prolonged health restrictions that have caused a performance deficit. Possible interventions are discussed and agreed upon in a round table consisting of a supervisor, an HR specialist, a company physician, a work council member, and—in some cases—an accommodation manager. Implemented workplace accommodations take three primary forms: (1) Transfer to another more suitable workplace, For instance, owing to shoulder problems and subsequent surgery, an employee was no longer capable of fulfilling the overhead tasks in his or her current workplace. Therefore, the employee was transferred to a workplace without overhead work. (2) Ergonomic adjustment of the original workstation. For instance, due to an irreversible damage of a hip joint, an employee was no longer capable of performing his or her current manufacturing task, which required permanent standing and walking. Therefore, the workplace was adjusted by providing a moveable seat and by rearranging the positions of the manufacturing components. Thus, work tasks could be fulfilled equally efficiently while seated. (3) Changes in working conditions. For instance, owing to several herniated disks and a subsequent spinal fusion of the lower back, an employee was no longer capable of working at any of the eight workstations of his or her team (job rotation). Therefore, the employee was excluded from job rotation and was permanently assigned to a single workspace in his or her former team. Because there was only low back strain, he or she was able to fully perform the required task.

### Participants

Our study sample consisted of 92 randomly selected accommodation recipients. All participants were workers in the factory, working in different steps of the manufacturing process, with most jobs being physically demanding. Of these 92 invited employees, 73 finally participated in our study (79% response rate). These employees had different kinds of health restrictions, all of which had impeded them to perform their initial jobs successfully in the past. As a consequence, all participants of our study were currently part of the company's job accommodation program or had undergone the accommodation process in the past two years. Participants' age ranged between 18 and 58 years; mean age was 46.6 years (6.6 years above the manufacturing department's mean age). The majority of participants (90.6%) was male. On average, participants were transferred 1.8 times during their accommodation phase. The mean process duration was 11.0 months.

### Data collection

We conducted 15 focus group interviews applying a variation of the *nominal group technique* (Delbecq et al., 1975). In this form of groupwork, participants individually generate ideas in response to specific guiding questions. We chose this technique in order to receive a wide variability of non-biased responses in a time efficient manner. Additionally, the topic of workplace accommodations was considered a sensitive issue that might not be easy to talk about. This was aggravated by the fact that most participants did not know each other prior to the group interview. The nominal group technique has been proven effective in studies with client populations dealing with similarly sensitive topics such as severe physical disabilities (e.g., Elliott and Shewchuk, 2002). In our study, participants were first welcomed by a company's accommodation process manager who ensured absolute confidentiality, introduced the independent focus group facilitators, and then left the room. The two facilitators then provided information about the 2 h procedure to follow. After filling out a short demographic questionaire, the participants were asked to individually write down their ideas and impressions, guided by the following two questions: (1) *What types of positive aspects occurred during your accommodation process?* (green metaplan cards) (2) *What types of negative aspects or problems occurred during your accommodation process?* (red metaplan cards). Afterwards, all notes were shared with the group, collected and roughly clustered on a board by the facilitator. This procedure helped ensure that participants could speak up if their card content was misunderstood. Finally, there was a group discussion on the generated aspects during which participants were also encouraged to write down any new aspect arising from the discussion.

Owing to very strict laws and regulations governing data protection, especially concerning health-related topics in this company and in Germany in general, we were not permitted to audiotape or videotape the nominal group sessions. We used the metaplan cards written by the participants as subject to the qualitative analysis. In cases where comments on the metaplan cards were too short or hard to understand, the facilitator directly asked the participant for further explication during the discussion phase; the comments were then added by the facilitator, using original terms. Following the sessions, the facilitators documented the results by taking photographs. Both researchers then jointly wrote a session reflection summarizing their observations (e.g., group atmosphere, displayed emotions, and key discussion topics).

### Analysis

For the analysis, we adopted a procedure in between an inductive and a deductive approach. *Template analysis* (King, 1998) is a suitable way to build on existing theories; at the same time, it also leaves enough space for unanticipated themes emerging from the data. The method starts out with an a priori template of codes, expands it while analyzing the data, arriving at a final template (Crabtree and Miller, 1992). In this way, the approach enabled us to verify and advance the theoretical framework developed above. The template analysis approach has generally been proven effective in other organizational studies dealing with similar topics such as leaders' negative emotions (Lindebaum and Fielden, 2011) or tensions and challenges associated with diversity and inclusion management (Donnelly, 2015).

All metaplan cards from the nominal group sessions were digitized and imported into MaxQDA. Following the approach by Randall et al. (2007), the two researchers who had been present during the group sessions interactively worked together on the process of coding each comment. While comparing the metaplan cards to the initial theoretical framework, there were two possible coding outcomes (Randall et al., 2007): Either the segment of text was coded in line with the theoretical framework (Figure 1), or the template had to be modified or supplemented. In multiple iterative steps, each separate metaplan content was re-examined with the revised template until a final template was reached. To enhance the coding's reliability, two further researchers who had not been involved in the group interviews independently repeated the coding process, using the final template. With an agreement over 90%, the two resulting templates were very similar, apart from minor exceptions. These exceptions especially referred to the names given to the unexpected categories that came up during the data analyses. The final category names for unexpected topics were then derived by means of a group discussion among all researchers.

Beyond the negative aspects of accommodation processes which were coded into the proposed framework, we also analyzed positive aspects. Here, we used the neutral overarching categories *interpersonal experiences, process-related experiences*, and *accommodation recipients' affective reactions*. Subcategories within these broad themes were generated in an exploratory manner while working through the data.

## Results

In sum, we collected 285 metaplan cards, of which 218 (76%) referred to the question concerning issues and problems during the accommodation process; 17 metaplan cards had to be excluded owing to unrelatedness to the guiding questions or incomprehensibility. Some participants noted several content aspects on one single card; thus, the 268 metaplan cards subject to the analyses resulted in 276 codings (208 negative and 68 positive codings). The final template, including the frequencies of the codes referring to our negative guiding question, is shown in Table 1, while the positive counterpart is provided in Table 3. Exemplary quotes for the negative aspects of job accommodations can be found in Table 2, while quotes capturing the positive aspects of job accommodations can be found in Table 4.

**Table 1:** Final categorization of recipients' negative experiences (the percentage of total negative codings appears in parentheses).

	**Category**		**No. of times mentioned**		**Sum**
**A**.	**Interpersonal experiences**				**109 (52.4%)**
A.1	Lack of social support			38 (18.3%)	
	By coworkers		6 (2.9%)		
	By supervisors/other stakeholders		21 (10.1%)		
	Unknown referent		11 (5.3%)		
A.2.	Discrimination, bullying, and maltreatment			50 (24.0%)	
	By coworkers		23 (11.1%)		
	Discrimination and Bullying	4 (1.9%)			
	Envy	5 (2.4%)			
	Conflict Old vs. Young	14 (6.7%)			
	By supervisors/Other stakeholders		20 (9.6%)		
	Unknown referent		7 (3.4%)		
A.3.	Lack of communication or information			21 (10.1%)	
**B**.	**Process-related experiences**				**81 (38.9%)**
B.1.	Lack of participation			2 (1.0%)	
B.2.	Lack of accommodation effectiveness			14 (6.7%)	
B.3.	Process duration			23 (11.1%)	
B.4.	Lack of accommodation possibilities			13 (6.3%)	
B.5.	Feeling of dehumanization			5 (2.4%)	
B.5.	Other process-related problems and conflicts			24 (11.5%)	
**C**.	**Accommodation recipients' affective reactions**				**18 (8.7%)**
C.1.	Uncertainty			5 (2.4%)	
C.2.	Stress and strain			13 (6.3%)	
					208 (100%)

**Table 2:** Quotes of recipients' negative experiences.

	**Category**	**Quote**
**A**.	**Interpersonal experiences**	
A.1	Lack of social support	
	By Coworkers	“Coworkers do not show understanding, especially when the disability is not directly visible.”; “Lack of sympathy on the part of my coworkers.”
	By supervisors/other stakeholders	“I had to look for a new workplace myself, without support of my supervisor.”; “My supervisor does not care about how employees with disabilities feel.”
	Unknown referent	“I have never been taken seriously with my disability.”; “I had to handle the accommodation on my own, no help.”
A.2.	Discrimination, bullying, and maltreatment	
	By coworkers	
	Discrimination and bullying	“Coworkers: Bullying!”; “Colleagues sneer at me.”
	Envy	“Some of my colleagues are envious of my new workplace and try to give me even more work to do.”; “Envy of others” (healthy) colleagues
	Conflict old vs. Young	“With my performance I have to compete with younger, fitter employees, this is not okay.”; “‘Easy’ workplaces are occupied by young employees.”
	By supervisors/Other stakeholders	“My supervisor threatened to fire me.”; “Supervisor talks to me in an insolent and insulting way.”
	Unknown referent	“I was called a liar.”; “False accusations.”
A.3.	Lack of communication or information	“Unknown point of contact – didn't know who to turn to.”; “Arrangements between HR, work council, supervisor, company physicians: poor communication.”
**B**.	**Process-related experiences**	
B.1.	Lack of participation	“Round table: no result, no participation.”; “No participation/voice with my supervisor.”
B.2.	Lack of accommodation effectiveness	“I must perform tasks I actually should not do at my new workplace.”; “Doctor's restrictions are disrespected.”
B.3.	Process duration	“It all took very long.”; “Too long (two years).”
B.4.	Lack of accommodation possibilities	“Not enough workplaces suitable for accommodation recipients.”; “No ‘easy’ workplaces available.”
B.5.	Other process-related problems and conflicts	“I was downgraded in my wage group.”; “New shift (carpool).”
**C**.	**Accommodation recipients' affective reactions**	
C.1.	Uncertainty	“Uncertainty about whether one is allowed to stay at this workplace.”; “Uncertainty.”
C.2.	Stress and strain	“Stress through too many job transfers.”; “Performance pressure from above.”
C.3.	Feeling of dehumanization	“Individual problems are not noticed—people are seen as numbers.”; “Quality and quantity; people are forgotten.”

**Table 3:** Final categorization of recipients' positive experiences (the percentage of total positive codings appears in parentheses).

	**Category**	**No. of times mentioned**		**Sum**
**A**.	**Interpersonal experiences**			**30 (44.1%)**
A.1	Social support		28 (41.2%)	
	By coworkers	8 (11.8%)		
	By supervisors/other stakeholders	20 (29.4%)		
A.2.	Communication or information		2 (2.9%)	
**B**.	**Process-related experiences**			**37 (54.4%)**
B.1.	Participation		4 (5.9%)	
B.2.	Effectiveness of accommodation		24 (35.3%)	
B.3.	Short process duration		7 (10.3%)	
B.4.	Others		2 (2.9%)	
**C**.	**Accommodation recipients' affective reactions**			**1 (1.5%)**
C.1.	Certainty/Job retention		1 (1.5%)	
				68 (100%)

**Table 4:** Quotes for recipients' positive experiences.

	**Category**	**Quote**
**A**.	**Interpersonal experiences**	
A.1	Social support	
	By coworkers	“Help and support from colleagues”; “Accepted by coworkers: we get along well”
	By supervisors/other stakeholders	“My supervisor is supporting me”; “Former supervisor personally fought for my transition”
A.2.	Communication or information	“Good communication between the responsible parties in the process”
**B**.	**Process-related experiences**	
B.1.	Participation	“My proposals for the configuration of the new workplace were considered”; “Autonomy in designing my workplace”
B.2.	Effectiveness of accommodation	“I found an appropriate workplace”; “The new workplace is good”; “Less physical strain now”
B.3.	Short process duration	“All worked out very quick”; “Quick transfer”; “Immediate action”
B.4.	Others	“Funding by the German Federal Pension Insurance was possible”
**C**.	**Accommodation recipients' affective reactions**	
C.1.	Certainty/Job retention	“I know that I can keep this workplace until I retire (safety)”

### Negative interpersonal experiences (109 codings, 52% of total)

#### Lack of social support (38 codings, 18% of total)

Participants referring to a lack of social support stated for instance that they had to organize their job accommodation without the help of supervisors or central functions such as HR. Other support-related comments concerned the lack of consideration and appreciation for accommodation recipients and a lack of understanding for the person's disability. *Supervisors and other stakeholders* (HR department, company physicians) were more often identified as sources of a lack of support *(21 codings, 10% of total)* than *coworkers (11 codings, 5% of total)*.

#### Discrimination, bullying, and maltreatment (50 codings, 24% of total)

*Coworkers* were mentioned slightly more frequently as sources of discrimination, bullying, and maltreatment *(23 codings, 11% of total)* than *supervisors and other stakeholders (20 codings, 10% of total)*. Discriminatory behavior from supervisors was especially characterized by insulting and disrespectful communication to the respondents. Participants also felt threatened and placed under pressure (e.g., threat of dismissal). In statements referring to *coworkers*, participants reported false accusations, being called liars, or being derided (*4 codings, 2% of total)*. We were also able to identify codings referring to *envy by coworkers (5 codings, 2% of total)*. That is, coworkers envied accommodation recipients for having received more favorable job conditions (e.g., “some of my colleagues are envious of my new workplace and try to give me even more work to do”). Additionally, owing to a high number of similar comments in the category *discrimination, bullying, and maltreatment*, a new subcategory arose from the data analysis that we did not expect a priori. We found that quite a few participants mentioned statements we refer to as the *conflict old vs. young (14 codings, 7% of total)*. Such statements criticized equal performance expectations, irrespective of age and competition for desirable workplaces (e.g., “with my performance, I must compete with younger, fitter employees; this is not ok”). Some participants claimed that younger employees were favored by supervisors or other stakeholders (e.g., “young workers are preferred”). Other statements also implied negative attributions towards younger employees (e.g., “young employees are too sniveling”).

#### Lack of communication or information (21 codings, 10% of total)

Participants often criticized the absence of a dedicated contact person concerned with the accommodation process and a lack of feedback on the handling of their individual case. They also stated that they were not heard by supervisors and that communication between process stakeholders was poor.

### Negative process-related experiences (81 codings, 39% of total)

#### Lack of participation (2 codings, 1% of total)

The lack of possibility to participate in the accommodation process was criticized only twice. From our initial framework, we expected a significantly larger number of comments.

#### Lack of accommodation effectiveness (14 codings, 7% of total)

Participants criticizing the lack of situational improvement stated especially that their health restrictions (e.g., no overhead work) were not respected or that no accommodation measures had been taken at all.

#### Process duration (23 codings, 11% of total)

Participants claimed that they had waited a long time until the final implementation of an accommodation, or that they had been transferred too many times during the process.

#### Lack of accommodation possibilities (13 codings, 6% of total)

The major aspect was the absence of “easy” (i.e., less strenuous) work for accommodation recipients. Many participants also criticized that such jobs were increasingly outsourced or combined with additional tasks.

Data analysis resulted in two new categories classified as unexpected process-related issues. We called them *feeling of dehumanization (5 codings, 2% of total)* and o*ther process-related problems and conflicts (24 codings, 12% of total)*. Statements classified as *feeling of dehumanization* refer to participants' perceptions of being “treated like numbers” and not being acknowledged as individuals but being reduced to one's work output (e.g., “Individual problems are not noticed. People are seen as numbers”). The second new category, *other process-related problems and conflicts*, arose because some metaplan content did not match the existing codes. Issues raised in this category were mostly very specific individual disadvantages in the accommodation process (e.g., “loss of carpooling opportunity due to shift change”), or perceptions that could count as “single opinions” not mentioned by other participants.

### Negative accommodation recipients' affective reactions (18 codings, 9% of total)

#### Uncertainty (5 codings, 2% of total)

Most statements in this category referred to doubts and uncertainty about the future, especially concerning job security.

#### Stress and strain (13 codings, 6% of total)

On the one hand, participants reported stress and strain emerging from the job accommodation itself, especially resulting from a high number of job transfers and treatment by other process stakeholders. On the other hand, respondents also emphasized that they felt stressed by high workloads in their teams and the pressure to perform. Besides the codings for affective reactions, also other codings classified into different categories were emotionally charged. Some codings reflected feelings of concern (e.g., “it's a matter of sink or swim”), others revealed a bitter, cynical tone (e.g., “more and more people in suits and less and less workers”), some sounded disappointed and sad (e.g., “nobody asks you how you feel”). These findings were also strongly supported by the impressions gained during the nominal group sessions and summarized in the session reflections. Researchers observed that some participants reacted very emotionally when talking about their accommodation process, which was revealed by intonation, facial expressions, and body language.

### Positive aspects of the accommodation process

Although this study's focus lies in examining negative experiences during accommodation processes, participants were likewise asked to report about their positive experiences. Overall, 68 metaplan cards referred to such positive experiences during the recipients' accommodation process. These positive experiences were analyzed separately and represent 24% of all codings. The final template, including frequencies of the codes concerning the positive guiding question, are displayed in Table 3.

#### Positive interpersonal experiences (30 codings, 44% of total)

In contrast to the preceding analysis of negative aspects, interpersonal experiences were not mentioned most frequently but were exceeded by process-related experiences. The major interpersonal strength was *social support from supervisors and other stakeholders (20 codings, 29% of total positives)*, with most statements referring to a fairly practical, instrumental kind of support (e.g., “my former supervisor personally fought for my transition”).

#### Positive process-related experiences (37 codings, 54% of total)

Overall, positive codings were most frequently referring to process-related experiences. Thereby, the most frequently mentioned process-related strength was *accommodation effectiveness (24 codings, 35% total positives)*. Statements in this subcategory mostly referred to workplace aspects that had improved owing to the accommodation (e.g., “less physical strain now”).

#### Positive accommodation recipients' affective reactions (1 coding, 1% of total)

Compared to the preceding analysis of negative aspects, positive affective reactions were extremely rare. In fact, only one positive statement was provided that referred to the certainty of knowing that he or she can keep the accommodated workplace until retirement.

## Discussion

Prior research on workplace accommodations has provided important insights for scholars and organizations to better understand how individuals and organizations request, manage, and perceive workplace accommodations. Still, some important gaps in the literature remained; our study sought to address these. Most importantly, we sought to develop a systematic view of job accommodations by focusing on recipients' negative experiences during and after the job accommodations. In contrast to prior work, our study investigated job accommodations in a later chronological phase (i.e., after being granted and implemented) and in a context in which supervisors (instead of employees) initiate the job accommodations.

In sum, the high number of negative statements made in the focus group interviews indicates that negative experiences are a substantial part of accommodation processes (Baldridge and Veiga, 2006; Davison et al., 2009). On the one hand, the analysis of accommodation recipients' positive experiences shows that job accommodations tend to solve practical problems employees are struggling with; on the other hand, accommodations seem to generate a wide range of other challenges that have not yet received the attention they deserve (Colella and Bruyère, 2011). Notably, we consider this company to be a best practice example in systematically dealing with employees with disabilities and their related health impairments. Therefore, it is even more remarkable that we were able to identify this wide range of negative experiences among accommodation recipients in this company. The findings highlight the relevance of this topic, since even very well managed accommodation processes can lead to negative experiences and reactions among employees. Interestingly, most of these negative experiences do not stem from aspects directly related to the accommodation process—instead, accommodation success largely seems to depend on the social environment of the employees at work, especially their relationships with coworkers and supervisors.

Our findings also support our proposition that there are important similarities between individual accommodation processes and broader organizational change processes. Comparable to an organizational change process (Kiefer, 2005), an accommodation can be interpreted as a critical, affect-laden change experience that is associated with feelings of stress, strain, and uncertainty. In addition, some supportive conditions known from organizational change settings seem to also be applicable to accommodation contexts. Especially a supportive environment (Vakola and Nikolaou, 2005) seems to be significant for accommodation recipients, since our analysis revealed many corresponding complaints. Other supportive conditions known from organizational change research that became apparent in the accommodation process are communication and information (Lewis and Seibold, 1998) or change effectiveness (Reichers et al., 1997). In the following, we will discuss the implications of the study's main findings—including unexpected findings that arose during data analyses—and derive practical recommendations for organizations.

### Negative interpersonal and process-related experiences during accommodation processes

The most frequently reported negative experiences referred to interpersonal issues, especially perceived discrimination, bullying, and maltreatment. Surprisingly, while we initially expected coworkers to primarily engage in discrimination, bullying, and maltreatment, participants often reported of supervisors being the sources for such hostile behavior, almost as often as coworkers. This *vertical aggression* (Cortina et al., 2001) carried out by authorities indicates that leaders might have strong considerations against employees with disabilities that become noticeable during the accommodation process. Furthermore, we found participants reporting about conflicts between old and young employees during data analysis. On the one hand, the statements suggest that these participants were confronted with phenomena such as stereotyping and perceived age discrimination (ageism; Rupp et al., 2006; Bal et al., 2011). This might be explained by the *poor performance* and *resistance to change* stereotypes (Posthuma and Campion, 2009; Kunze et al., 2013), which are often held about older employees. However, on the other hand, participants expressed negative stereotypes against *younger* employees themselves, reinforcing that ageism can occur in both directions—against old and young employees (Kunze et al., 2011).

As compared to interpersonal issues, process-related issues were less frequently mentioned as a negative experience, the most common problem being long process duration. Some participants also criticized insufficient accommodation effectiveness; however, the positive evaluations revealed that *improvement of the situation* was also considered to be the major strength by most participants. Interestingly, perceptions of social support from coworkers and supervisors were very heterogeneous, with 38 negative comments and 28 positive comments. Since positive and negative aspects were raised by different employees, we conclude that these experiences are highly individual. In particular, supervisors and work groups seem to differ concerning the support level they provide toward their followers/colleagues with health restrictions. This important finding clearly calls for interventions such as awareness and leadership trainings offered throughout the organization.

Concerning process-related aspects, our data analysis also revealed an unexpected finding, i.e., the *feeling of dehumanization* that participants experienced. The concept of dehumanization is not entirely new to the research, though. Dehumanizing others, i.e., denying others “qualities associated with meaning, interest, and compassion” (Barnard, 2001, p. 98) is a phenomenon that sometimes affects the perception of people with disabilities (Haslam, 2006). Moreover, dehumanization to the extent that a person is seen as “object- or automaton-like” (Haslam, 2006, p. 258) is more likely to occur in an environment that is dominated by technology, just like in our research setting in the manufacturing industry.

### Employee-initiated vs. supervisor-initiated job accommodations in a non-US setting

Another goal of our study was to examine job accommodations in a non-US setting. It is striking that most prior studies have focused on accommodation requests by employees (e.g., Baldridge and Veiga, 2006). This seems to be due to the fact that accommodation requests represent a bottleneck in the US ADA-based system, which is why research on this topic is crucial. Nevertheless, in contrast to the US context where a person with a disability is usually expected to initiate the accommodation process, in our study's context, accommodations are initiated by supervisors based on prior performance deficits. We believe that the experiences that participants reported in the present study correspond to experiences of accommodation recipients in different organizational contexts. However, the fact that the accommodations in this study context had been initiated by the supervisor (instead of self-initiated) might be important to consider while interpreting our findings. As an example, the fact that many participants criticized a lack of support from their supervisors might seem surprising, given that those supervisors actively initiated these workplace accommodations for their employees. However, as we know from the field of organizational change, affected employees' acceptance of change largely depends on the sense of agency, competence, and internal control that they feel during the process (Amiot et al., 2006; Oreg et al., 2011). That way, some participants in our study reported that they would have wished to be more deeply involved in the accommodation process by their supervisors, to be asked for their individual needs and requirements, etc. Thus, since the participants in our study might perceive the process as rather externally imposed, they might experience the accommodation more negatively as compared to self-initiated accommodations. At the same time, some of the experiences defined in our model might be even stronger in the context of employee-initiated accommodation processes, such as bullying by coworkers (being called a liar etc.). To sum up, while our study is non-comparative, was conducted in only one German organization, and thus does not allow for causal interpretations, our results still seem to imply that supervisor-initiated accommodation processes might be particularly prone to critical employee reactions, especially if they are not backed by increased levels of social support demonstrated by the leader.

### Practical implications

An important contribution of our study is to derive success factors helping organizations to implement accommodation processes more effectively. Based on the comparability of change and accommodation processes, we propose that accommodation managers can learn from the comprehensive knowledge available in change management literature. Just as organizational change processes, job accommodations must also be actively managed and accompanied. Research has suggested many critical factors for successful change reaching from “soft” factors (e.g., employee motivation, leadership styles, or corporate culture) to “hard” factors (including project evaluation, project teams' skills, clear communication, and a limitation of additional workload) (Sirkin et al., 2005). We will now explicate some factors that appear to be most relevant for job accommodation processes, based on our study results.

#### Sufficient communication and information

During organizational change, goals and purposes of change must be clearly communicated (Elving, 2005) for employees to feel better prepared and able to deal with change (Bordia et al., 2006). Moreover, communication reduces uncertainty and cynicism (Schweiger and DeNisi, 1991) and increases perceived procedural justice, trust, and commitment (Gopinath and Becker, 2000). Likewise, we recommend that, during the accommodation process, accommodation recipients should receive detailed and pro-active information about their future work environment, the reasons for specific chosen accommodations, and how the process will proceed. In addition, supervisors and other responsible parties should receive dedicated training on the accommodation process in order to increase their process knowledge and to improve communication quality.

#### Sufficient resources

Concerning change initiatives, necessary resources must be made available, including skillful personnel and work capacity (Amiot et al., 2006; Rafferty and Griffin, 2006). Appropriately skilled change managers are crucial if change goals are to be met. Equally, we recommend that job accommodation processes should be ideally accompanied by an accommodation manager responsible for the planning and implementation of the process. This should also allow a fairly flexible and individualized treatment of every unique case. Additionally, accommodation processes might require further resources, for instance, training of different stakeholders or the redesign and accommodation of individual workplaces.

#### Sufficient monitoring

Successful change initiatives are characterized by frequent project reviews that help identify problems in early stages, making corrective action possible (Sirkin et al., 2005). Likewise, looking at the high individualization of problems during job accommodations, there should be a sufficient monitoring and feedback mechanism indicating whether or not an accommodation was successful. Especially by asking accommodation recipients about their satisfaction with the process, future accommodations can be improved.

#### Inclusive climate

Finally, issues such as discrimination, bullying, being envied, and conflicts between older and younger employees seem to arise often in the accommodation context. In this regard, recent research in the diversity domain suggests that creating an inclusive climate (Nishii, 2013; Dwertmann and Boehm, 2016) might be a key to success. Inclusion is defined as the “degree to which an employee perceives that he or she is an esteemed member of the workgroup through experiencing treatment that satisfies his or her needs for belongingness and uniqueness” (Shore et al., 2011, p. 1265). Therefore, promoting an inclusive climate that actively promotes diversity in the workgroup might create a culture in which all employees are equally valued—irrespective of their age or disability status.

#### Individualized leadership

Our findings show that many complaints raised by study participants referred to very unique issues (category other process-related problems and conflicts), suggesting that many problems during accommodation processes are highly individual and depend on every employee's unique circumstances. As a consequence, supervisors should individualize their leadership behavior to the unique needs and requirements of every accommodation recipient, instead of applying a “one-size-fits-all” approach. Supporting this view, Kensbock and Boehm (2015) have shown that individualized consideration, as part of an overall transformational leadership style, can be a successful strategy in fostering the health and job performance of employees with disabilities.

### Limitations and future research

A first potential limitation refers to the metaplan data we used for our qualitative analysis. It is certainly more recommendable to record interview data via audio or video in order to draw from a more extensive and rich dataset. However, owing to organizational restrictions, we were unable to do so. We did not have the impression that the data were not suitable for template analysis, though, since only a small number of metaplan cards had to be excluded due to incomprehensibility. At the same time, we experienced high openness when discussing these sensitive issues. Participants highly valued the fact that we guaranteed full anonymity and that we conducted no audio or video recording. Our claim of anonymity was more credible and less risky to trust in without taping. Otherwise, in our view, the willingness to talk about health-related issues, to discuss personal impairments and limitations, and to openly criticize their employer, supervisors, and other staff would have been significantly lower. This potential increase in data reliability and validity might compensate for the loss in data richness. We also made sure that we understood the metaplan cards in the right way by letting the participants explain their cards and clustering them in front of the group. This procedure gave particpants the space to speak up if their card's wording seemed to be misunderstood by the facilitators or other participants. This led to an additional validation of the data. Moreover, the session reflections provided additional data that was considered when interpreting the results.

A second limitation refers to the generalizability of our findings. Our study was conducted in a research context that might show special features concerning accommodations: In the production industry, employees work together in highly standardized, automated, and interdependent ways. Future research should apply our framework to other work contexts to check applicability. Future research should thus set out to generalize the proposed framework to other contexts and industries, especially looking at industries in which the degree of interdependence is not as high (e.g., office-dominated work). Furthermore, since our study was conducted in only one organization, future research should test the generalizability of our findings across organizational boundaries. In particular, organizational culture might play a crucial role in determining how accommodation processes are implemented by the management and perceived by the employees.

Third, one could ask whether the negative perceptions reported by the accommodation recipients might be driven by the health restriction itself, which might have negatively affected their job satisfaction. Indeed, there is empirical support for a negative relationship between sickness and job satisfaction (Faragher et al., 2005; Pagán and Malo, 2009). However, other research has shown that employees with disabilities are not per se less satisfied with their jobs, but that it depends largely on the organizational context, including the flexibility to provide suitable accommodations (Baumgärtner et al., 2015) or the organizational culture (Schur et al., 2009). Moreover, even if it might be that these negative perceptions cannot be exclusively attributed to the accommodation process itself, they might still have a negative impact on many important outcomes, such as commitment and turnover. As studies have shown for related fields such as perceived discrimination, “employees' beliefs, whether or not they are consistent with reality, affect their behaviors” (Ensher et al., 2001, p. 53). In addition, we think that our approach to ask participants about *both* positive and negative aspects of the accommodation process gave them the opportunity to reflect upon the accommodation process in a well-balanced way, thus preventing an overly negative mindset affecting their judgments.

Finally, some of our study results might reflect workplace aspects that are not limited to employees with health-related issues or disabilities. For instance, non-impaired workers might also report that they feel insufficiently supported by colleagues or a supervisor, or that they perceive an increasing dehumanization of production practices. Therefore, subsequent studies might include the perspective of further stakeholders in the process, such as supervisors and coworkers, to gain a more complete picture of accommodation processes.

Above all, qualitative methods do not seek to provide a statistical generalization, but rather to produce descriptions that help to determine possibly contrasting and contradictory trends in social processes, in this case, within the experiences of accommodation recipients. In turn, this might help to expand and generalize theory as opposed to test theory. Therefore, in order to gain a more generalizable understanding of the individual issues arising from job accommodations, further research is necessary, which should also include quantitative methods. Nevertheless, we hope that our study contributes to a better understanding of job accommodation processes and provides a solid foundation for future research.

## Author contributions

JK acted as the lead author who collected the data and was responsible for data analysis and manuscript preparation. SB led the overall research project, was responsible for the research design and wrote parts of the manuscript. KB collected the data and assisted in data analysis and manuscript preparation.

### Conflict of interest statement

The authors declare that the research was conducted in the absence of any commercial or financial relationships that could be construed as a potential conflict of interest.

## Footnotes

^1^SGB IX (Sozialgesetzbuch IX [Social Security Statute Book IX]) of (2007). Rehabilitation und Teilhabe behinderter Menschen. Allgemeine Regelungen (Rehabilitation and inclusion of individuals with disabilities. General regulations). Essen: Fachverlag CW Haarfeld GmbH.
